# Viscoelasticity and histology of the human cartilage in healthy and degenerated conditions of the knee

**DOI:** 10.1186/s13018-019-1308-5

**Published:** 2019-08-13

**Authors:** Michael Seidenstuecker, Julius Watrinet, Anke Bernstein, Norbert P. Suedkamp, Sergio H. Latorre, Anastasija Maks, Hermann O. Mayr

**Affiliations:** 1 G.E.R.N. Tissue Replacement, Regeneration & Neogenesis, Department of Orthopedics and Trauma Surgery, Medical Center - Albert-Ludwigs-University of Freiburg, Faculty of Medicine, Albert-Ludwigs-University of Freiburg, Freiburg im Breisgau, Germany; 2 Schoen Clinic Munich Harlaching, Teaching Hospital of Paracelsus Medical University Salzburg, Salzburg, Austria

**Keywords:** Gonarthrosis, Articular cartilage, Biomechanical testing, Mapping, Indentation, Histology, OARSI score, AHO score

## Abstract

**Background:**

There are many studies on osteoarthritis, but only a few studies deal with human arthrosis, comparing the mechanical properties of healthy and diseased samples. In most of these studies, only isolated areas of the tibia are examined. There is currently only one study investigating the complete mapping of cartilage tissue but not the difference between instantaneous modulus (IM) in healthy and diseased samples. The aim of this study is to investigate the relationship between the biomechanical and histological changes of articular cartilage in the pathogenesis of osteoarthritis.

**Methods:**

The study compared 25 tibiae with medial gonarthrosis and 13 healthy controls. The IM was determined by automated indentation mapping using a Mach-1 V500css testing machine. A grid was projected over the sample and stored so that all measurements could be taken at the same positions (100 ± 29 positions across the tibiae). This grid was then used to perform the thickness measurement using the needle method. Samples were then taken for histological examinations using a hollow milling machine. Then Giemsa and Safranin O staining were performed. In order to determine the degree of arthrosis according to histological criteria, the assessment was made with regard to Osteoarthritis Research Society International (OARSI) and AHO scores.

**Results:**

A significant difference (*p* < 0.05) could be observed in the measured IM between the controls with 3.43 ± 0.36 MPa and the samples with 2.09 ± 0.18 MPa. In addition, there was a significant difference in IM in terms of meniscus-covered and meniscus-uncovered areas. The difference in cartilage thickness between 2.25 ± 0.11 mm controls and 2.0 ± 0.07 mm samples was highly significant with *p* < 0.001. With regard to the OARSI and AHO scores, the samples differed significantly from the controls. The OARSI and AHO scores showed a significant difference between meniscus-covered and meniscus-uncovered areas.

**Conclusions:**

The controls showed significantly better viscoelastic behavior than the arthrotic samples in the measured IM. The measured biomechanical values showed a direct correlation between histological changes and altered biomechanics in gonarthrosis.

## Background

In Germany, 187,319 first implantations of total knee replacements (TKA) and 24,940 revision operations of TKA knee were performed in 2016 [1]. Osteoarthritis is the most common cause of movement deficiency in old age [2, 3] and its prevalence continues to increase [4]. Osteoarthritis is expected to be the most common cause of disability in 2030 [5, 6]. Changing mechanical stress on the articular cartilage is essential for the function and health of the cartilage. Unphysiologic stress, however, can cause degeneration of the cartilaginous tissue as well as the subchondral bone and lead to osteoarthritis. Degenerative changes in the articular cartilage can even affect every second German citizen at the age of over 60 years [7, 8]. Degenerative changes are manifested in softening and continuous thinning of the articular cartilage to complete bone baldness, which is extremely painful for the patient in the late stage and requires therapeutic intervention [8]. In addition, there is a thickening of the subchondral bone [9, 10]. If a meniscectomy is performed, the risk of developing osteoarthritis increases later in life; the result is a 132-fold increase in TKA compared to the control [11]. The menisci double the contact surface of the joint to twice [12]. This distributes the joint pressure over a larger area. Also, the “leeway” on a possible position of the force resultants in the joint is considerably expanded. If the meniscus is removed, this free space for directional changes of the resultant will be significantly reduced. The result is an increasing burden on the cartilage. At present, there is a paradigm shift in the treatment of meniscal damage—from distance to meniscal maintenance or meniscal replacement [13]. For the understanding of osteoarthrosis and the development of scaffolds for meniscal and cartilage replacement, knowledge of the mechanical properties of articular cartilage is essential, in particular, the influence of morphological changes of the meniscus on the mechanical properties of the cartilage and vice versa. Cyclic loads influence the viscoelastic properties. So far, there are very few studies dealing with the characterization of the mechanical properties of human joint cartilage [14–16], most studies deal with animal samples [17, 18]. In most cases, the determination of the mechanical properties had been carried out by means of unconfined compression tests [15, 16, 19]. Often, cylinders were punched out of the tibiae [15, 20] and examined by unconfined compression [19]. In this type of experiment, it has not been possible to perform a mapping of the mechanical properties over the entire tissue. This approach was taken up and optimized by Deneweth et al. [16] so that a rough mapping was possible by punching out 21 samples distributed over the entire tibial plateau and measuring them using unconfined compression. Other authors like Thambyah et al. [21] tried to investigate the differences between meniscus-covered (m-covered) and meniscus-uncovered (m-uncovered) areas with one punched out cylinder each via unconfined compression. Furthermore, the tissue was no longer suitable for histological analysis due to compression damage. Sim et al. [22, 23] showed in their work that automatic mapping of soft tissues, such as articular cartilage and meniscus, with the Biomomentum Inc. (Montreal, Canada) is possible. The focus in the previous work [22] was, however, to compare the values measured with the Mach-1 to a newly developed measuring device Arthro-BST which non-destructively measured the mechanical and biochemical values of articular cartilage during knee surgery. But in contrast to the investigations of Sim et al. [22], the focus of our investigations was to determine a biomechanical and histological difference between arthrotic joints, removed during TKA, and the healthy control with the same age distribution. So far, a mechanical characterization of the viscoelasticity of the cartilage of degenerated joints in comparison to healthy joints based on an indentation mapping test and regarding histological changes was not published.

## Methods

The samples were tibial plateaus taken during the implantation of a joint replacement due to grade 4 osteoarthritis according to Kellgren and Lawrence [24]. Twenty-five (double sides, with only one defect site) samples and 13 (12 double-sided, one single-sided) controls were analyzed. The controls were obtained from body donors (ethics vote 305/10 of the ethics commission of the Freiburg University Medical Center). All samples were handled according to approved institutional ethics committee certificates. Before the measurements, the positions of the menisci were marked with a tissue marker on the tibiae to distinguish between m-covered and m-uncovered. Only then was the biomechanical examination performed. Areas with pure bone were not measured, to protect the multi-axial load cell (for soft tissue). Subsequently, the samples were taken for histological examination. Finally, the samples were stored at − 80 °C.

### Mechanical examination

#### Automated indentation mapping

**Fig. 1:**
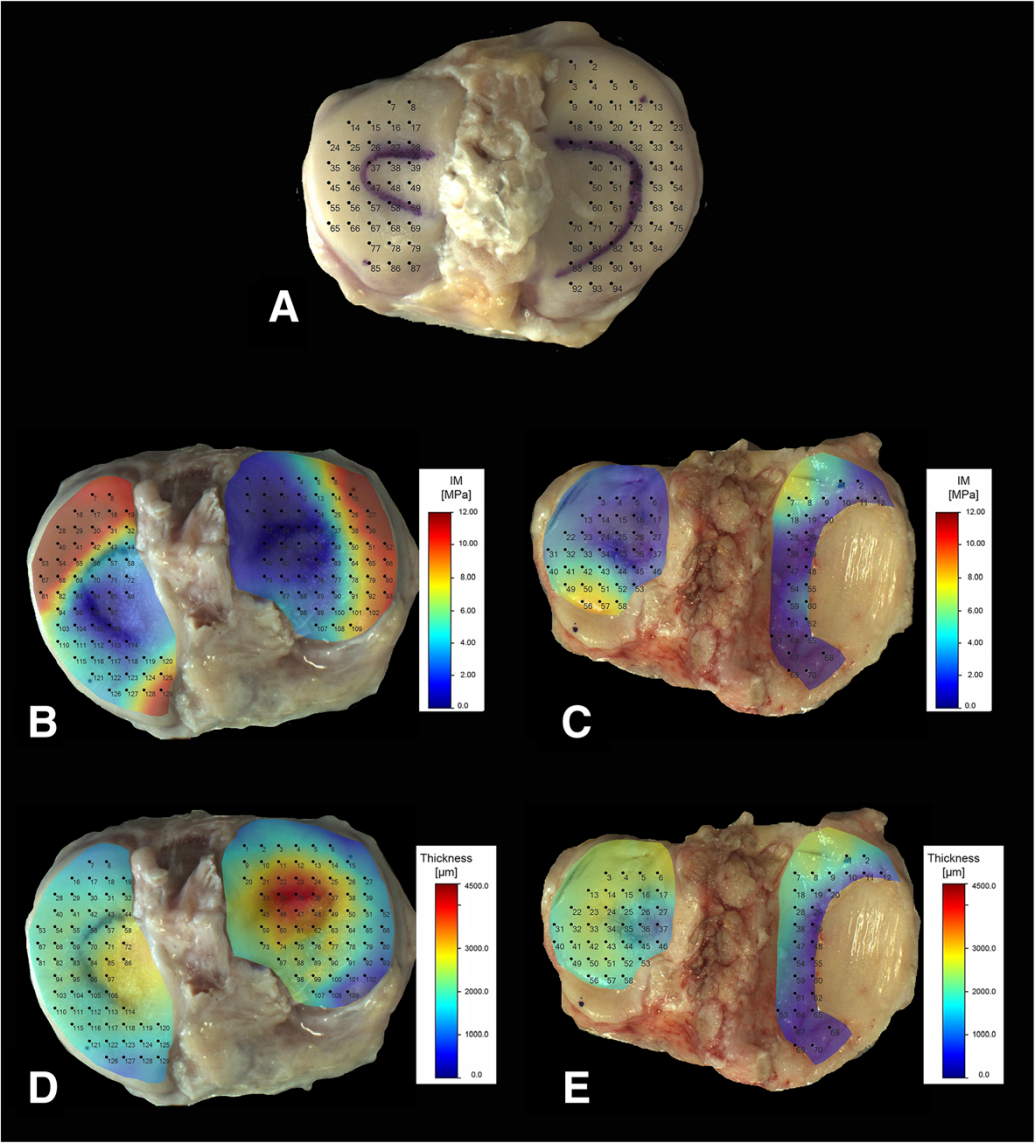
**a** Measuring points on the tibia, the distance between the points was 5 mm in both directions. Markings to distinguish the meniscus-covered and meniscus-uncovered areas. Comparison of surface mapping IM (**b** + **c**), thickness (**d** + **e**) of control (**b** + **d**) and specimen (**c** + **e**) with a large cartilage defect on the medial side. Measuring and mapping applied with Mach-1 (Biomomentum Inc.) in PBS at RT

The mechanical characterization was carried out by means of automated mapping [25] based on an indentation test (DIN EN ISO 14577) [26, 27]. For this, a Mach-1 Model V500css test device (Biomomentum Inc., Laval, Canada), a multi-axial load cell with 70N Model FTIFPS1 (ATI Industrial Automation, Apex, USA) and Newport Motion Controller ESP 301 (Newport, Irvine, USA), was used to examine the soft tissue and allow complete mapping of the tissues to be tested [25, 28]. First, the m-covered part was marked, in order to make a distinction of the areas later. Subsequently, the tibia resections were fixed by screw fixation on the sample holder. Thereafter, the determination of the measuring points on the sample followed. For this purpose, a measuring grid was projected onto the tibia (see Fig. 1). The distance between the individual points projected on the tibia was 5 mm in both directions.

Subsequently, the instantaneous modulus (IM) is determined in the form of an indentation method (DIN EN ISO 14577) by using a spherical indenter with 1 mm diameter. In contrast to previous studies [14, 20, 29–34], the entire tibia was examined. Therefore, at least 100 measurement points were equally distributed across the tibia. The contact criterion with the sample surface has been set to 0.1 N. The indentation amplitude was set to 0.3 mm by difference regulation, the indentation velocity to 0.1 mm/s, and the relaxation time to 10 s. To prevent the sample from drying out, the biomechanical measurements were carried out in PBS. Uplift forces were compensated via the Mach-1 testing machine. The remaining cartilage was examined except defect areas with pure bone, to protect the load cells of the Mach-1.

#### Automated thickness mapping

Thickness was mapped with the needle technique [35], as described before [36], by replacing the spherical indenter with a 27G × ¾″ intradermal needle (B.Braun, Melsungen, Germany). The following parameters were input into the Mach-1 Motion Software: stage velocity of 0.5 mm/s; contact criteria of 7 N, and stage repositioning of two times load resolution. The needle on the mechanical tester was directed vertically towards the sample at a constant speed until the cartilage surface was penetrated and the needle stopped at the subchondral bone edge [25]. The thickness mapping used the same measuring grid like the indentation experiment, in order to get the thickness information on the exact same positions on the tibiae.

#### Data processing

The findings were analyzed using the software Mach-1 Analysis Version 4.1.0.17 (Biomomentum, Montreal, Canada), Origin 2018 Professional (Origin Lab, Northampton, USA), and SPSS 23 (IBM, Armonk, USA). The evaluation methods used were according to Sim et al. [23]. Using automated thickness mapping results, the cartilage thickness was calculated at each position from the difference between the vertical position of the surface (where the load starts to increase) and the position of the cartilage/bone interface (corresponding to the first inflection point in the displacement/force curve). The IM at each position was obtained by fitting the load-displacement curve (with corresponding thickness and effective Poisson’s ratio of 0.5) to an elastic model for indentation according to Hayes et al. [37] (see the following equation).

$$ \mathrm{IM}=\frac{P}{H}\cdot \frac{1-{v}^2}{2 ak\cdot \left(\frac{a}{h}v\right)} $$

where *P* = load, *H* = indentation depth, *a* = radius of the contact region, *ν* = Poisson´s ratio, *k* = correction factor dependent on a/h and *ν*, and *h* = sample thickness.

### Histological examinations

After the biomechanical examinations, the tibiae were prepared for extensive histological examinations. For this purpose, cylindrical samples were taken from four areas (medial and lateral: m-covered, m-uncovered) by means of a cannulated reamer (DePuy Synthes, Zuchwil, Switzerland). The specimens were fixed in 4% buffered formaldehyde solution overnight. These samples were then decalcified for 2–3 weeks in decalcifying solution (EDTA, NaOH, pH 7.4), dehydrated in the tissue infiltration machine (Leica Biosystems, Nussloch, Germany), and then embedded in paraffin. The thin sections were made with a microtome (Leica Biosystems, Nussloch, Germany). These are then stained with Safranin O [38] and Giemsa. Microscopic assessment is based on two different scores. The OARSI score [39, 40] quantifies damages and changes to the cartilage and its structure whereas the score by Aho et al. [41] is focusing on the remodeling of the subchondral bone. Table 1 shows an overview of the different grades of the two scores.

**Table 1:** Overview of the different grades of the OARSI and AHO score

	Scores	
Grade	OARSI [39]	AHO [41]
0		No evident subchondral bone sclerosis, articular cartilage directly connected to the bone
1	Cartilage surface intact	Some subchondral sclerosis, bone volume increased, cartilage contact with the bone marrow
2	Cartilage surface discontinuity	Distinct increase in subchondral sclerosis and bone volume, fibrillation in subchondral bone, no contact of the bone marrow to the articular cartilage
3	Vertical fissures within the cartilage	Late-stage disease, severe subchondral sclerosis and massively increased bone volume, bone marrow distance from the cartilage increases
4	Erosion	
5	Denudation	
6	Deformation	

### Statistics

All values were expressed as mean ± standard error of the mean. Regarding the scores and all numerical values (if *n* < 5), statistical significance was tested non-parametrically primarily using the Mann-Whitney *U* test. Probability distributions of samples with *n* ≥ 5 were analyzed by a Kolmogorov-Smirnov test, and the Pearson-rho correlation test. Statistical significance was defined as *p* < 0.05. Based on the number of samples, it can be assumed that the samples are not normally distributed. Power calculation was performed in collaboration with the Institute of Medical Biometry and Statistics of the local university. A clinical relevant difference of the IM between the healthy control and the arthrotic specimen was determined with 2 MPa according to literature [19]. The SD was hypothesized with 0.5 MPa. To reach an alpha error level of 1%, at least 11 samples of each (specimen and control) were necessary. The online sample size and power calculator www.dssresearch.com were used. The calculated power for two different samples and two-tailed test with a difference of IM with 2 MPa, 25 samples and 13 controls, SD of 0.5 each, and alpha error level of 1% was calculated to be 100%.

## Results

On average, the patients were 72 ± 8 years old. The ratio of women to men in the study was 2:1. Only the remaining cartilage on the tibiae was examined. All the following calculations are only for the remaining cartilage without the pure bone parts (as you can see in Fig. 1).

### Biomechanical evaluation

For each tibia, depending on the size and the defect area, in mean 100 ± 29 (see Fig. 1a), different positions were measured.

#### Automated mapping

**Fig. 2:**
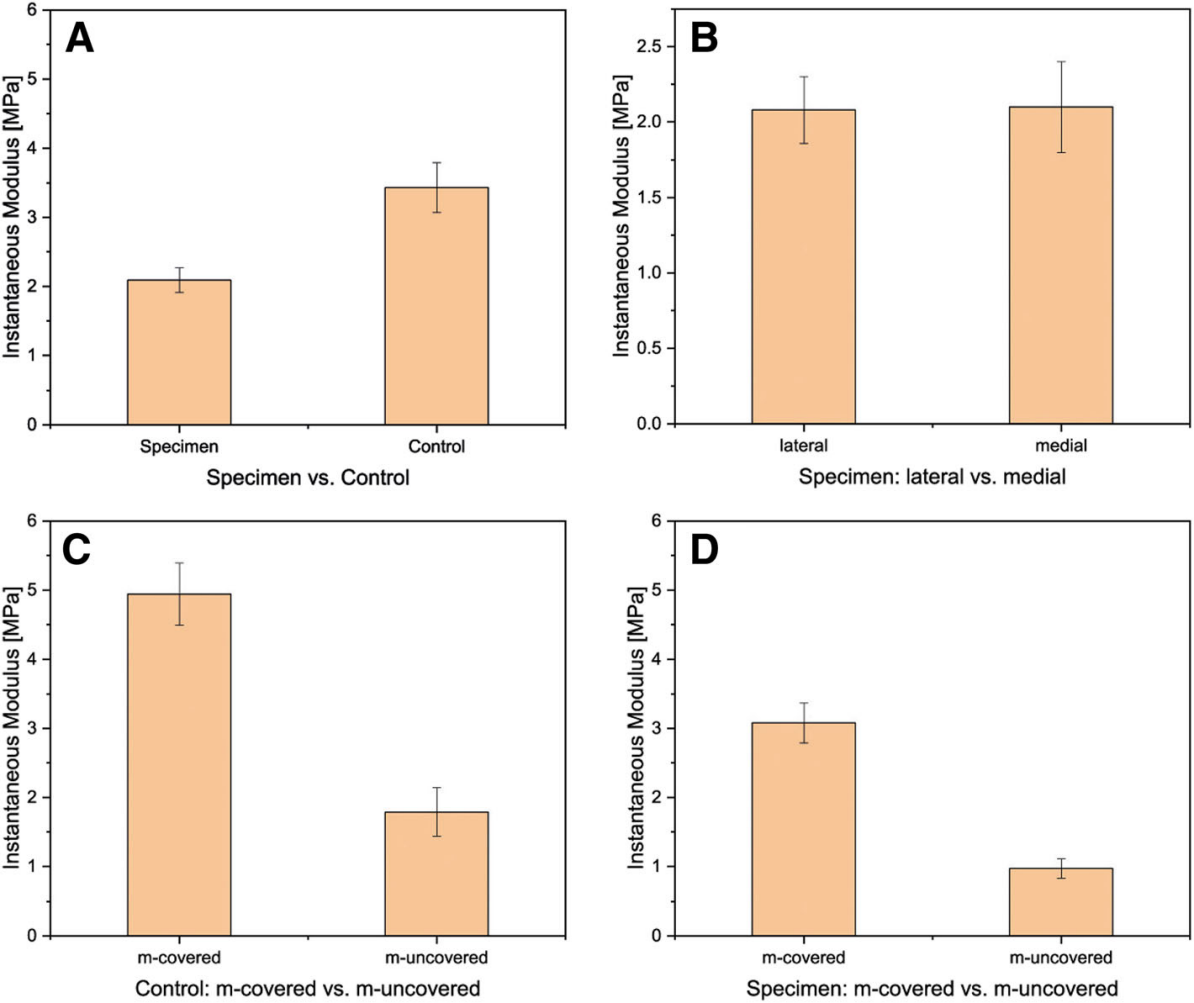
Comparison of instantaneous modulus for **a** specimen and control**:** specimen *N* = 25, control *N* = 13; *p* < 0.05. **b** Comparison of the IM and its location: medial vs lateral; medial *N* = 21, lateral *N* = 25. Comparison of the IM and its location: **c** cartilage covered vs not covered by meniscus (for medial and lateral); **d** specimen, m-covered *N* = 31, m-uncovered *N* = 30; right: controls, m-covered *N* = 25, m-uncovered *N* = 23

**Fig. 3:**
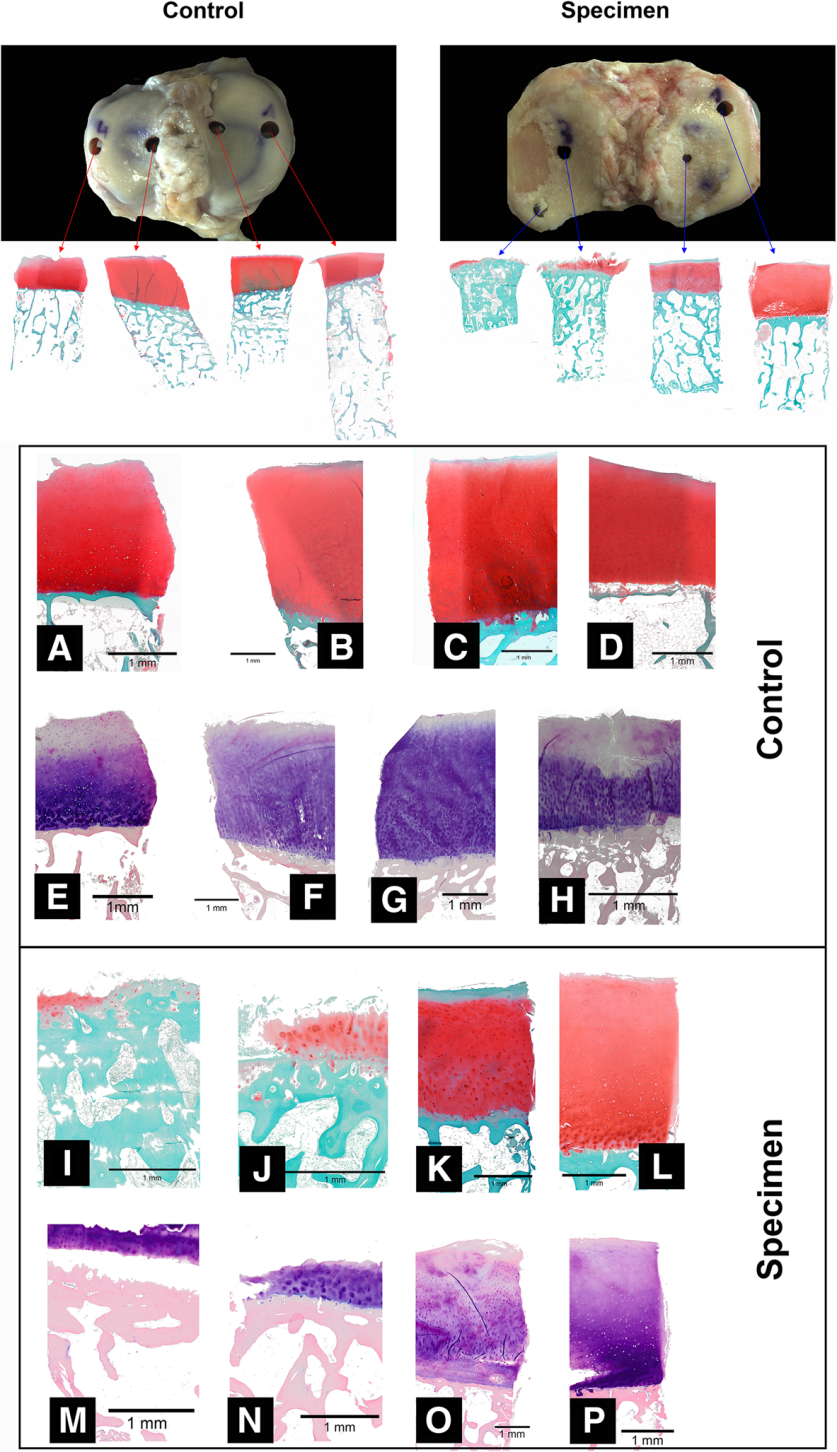
Overview of taken samples for histological examinations: control vs specimen. Comparison of Safranin O staining (**a**–**d**) and Giemsa staining: control group, **a**–**d** Safranin O staining and **e**–**h** Giemsa staining; specimen, **i**–**l** Safranin O staining and (**m**–**p**) Giemsa staining (enlargements of the images for control and specimen); black bar = 1 mm. Images were taken with Olympus BX-53

There was a significant difference in the measured IM with *p* < 0.05 between the controls at 3.43 ± 0.36 MPa and the specimen at 2.09 ± 0.18 MPa (see Figs. 1 and 2a). In comparison of the IM of the specimen lateral versus medial, no significant difference could be determined. The moduli were 3.17 ± 0.47 MPa for the lateral regions and 3.66 ± 0.55 MPa for the medial regions (see Fig. 2b). Comparing the m-covered with the m-uncovered areas of the arthrotic cartilage, a significant difference could be observed (see Fig. 2c, d). The m-covered cartilage areas had an IM of 3.08 ± 0.29 MPa and the m-uncovered 0.97 ± 0.14 MPa. In Fig. 3, the differences on surface mappings are shown for control and specimen. Especially, the m-covered areas were different (if present, in the right image in Fig. 1c, the m-covered cartilage is totally destroyed). The results of the automated mappings of the IM are summarized in Table 2.

**Table 2:** Overview of IM and max load across the tibiae

Sample	Mean ± SD	m-covered	m-uncovered	Lateral	Medial
Instantaneous modulus [MPa]					
Specimen	2.09 ± 0.18	3.08 ± 0.29	0.97 ± 0.14	2.08 ± 0.22	2.10 ± 0.30
Control	3.43 ± 0.36	4.94 ± 0.45	1.79 ± 0.34	3.17 ± 0.47	3.66 ± 0.55
Max load [N]					
Specimen	1.72 ± 0.24	1.55 ± 0.59	1.60 ± 0.70	1.71 ± 0.62	1.74 ± 0.38
Control	1.00 ± 0.11	1.51 ± 0.15	0.50 ± 0.10	0.88 ± 0.15	1.12 ± 0.17

#### Automated thickness mapping

Fig. 1 d and e clearly shows that the m-uncovered areas on the tibia have a significantly higher cartilage thickness than the m-covered areas. The cartilage thickness was compared to the controls, and the m-covered cartilage and the m-uncovered cartilage showed a significant difference of *p* < 0.05. The cartilage thickness of the samples was also significantly higher than that of the m-covered cartilage. The comparison of medial vs lateral cartilage thickness also showed a significant difference of *p* < 0.05 (see Table 3).

**Table 3:** Overview of cartilage thickness across the tibiae

Sample	Cartilage thickness [mm]				
	Mean ± SD	m-covered	m-uncovered	Lateral	Medial
Specimen	2.0 ± 0.07	2.07 ± 0.10	1.96 ± 0.15	2.15 ± 0.11	1.82 ± 0.06
Control	2.25 ± 0.11	2.14 ± 0.08	2.41 ± 0.22	2.39 ± 0.19	2.15 ± 0.12

### Histological examinations

Four cylinders were taken from each sample in m-covered and m-uncovered areas. Figure 3 shows an example of a comparison between a control and an arthrotic sample. It becomes clear that in comparison to the control, where all cartilage areas are intact, the arthrotic sample has only very small cartilage thicknesses and a defect site. In addition, the control sample shows very clearly that the areas covered by the meniscus have a lower cartilage thickness than the uncovered areas. This is no longer the case with the arthrotic sample. Only minimal cartilage thicknesses are left on the defect side at both sampling sites. The complementary side already shows changes in the cartilage. Figure 3a–p shows an enlarged view of the existing joint cartilage as Safranin O and Giemsa staining.

#### OARSI

**Fig. 4:**
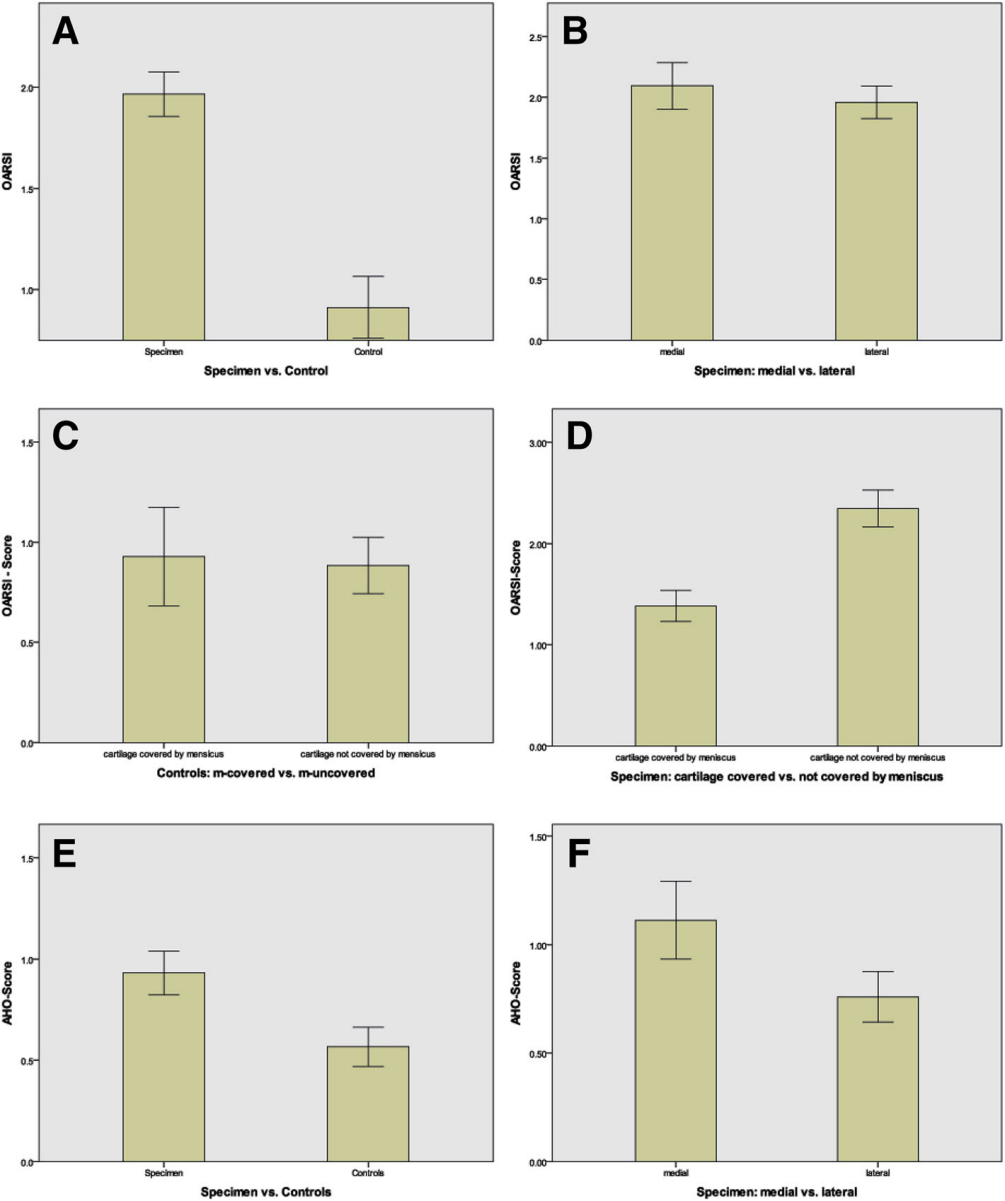
OARSI scores. **a** Specimen (*N* = 25) vs control (*N* = 13). **b** Specimen medial (*N* = 22) vs lateral (*N* = 25); m-covered vs m-uncovered (lateral and medial). **c** Controls. **d** Specimen; AHO score of **e** specimen (*N* = 32) vs control (*N* = 38). **f** Comparison of medial vs lateral

The OARSI score shows a significant difference between diseased (specimen) and healthy knees (control) as well as between m-covered and m-uncovered areas. If the affected population is considered alone, a significant difference (*p* < 0.001) between m-covered and m-uncovered areas can be observed. There are no differences in the OARSI score between the medial and lateral side (see Fig. 4). Furthermore, the values of the OARSI score correlate significantly negatively with the cartilage thickness (− 0.26 in Kendall-tau, − 0.11 in Pearson-rho) and more negatively in IM (− 0.37 in Kendall-tau, − 0.33 in Pearson-rho). A comparison of the OARSI and AHO scores is shown in Table 4.

**Table 4:** Comparison of different scores for specimen and control

		Median ± SD	m-covered	m-uncovered
OARSI	Specimen	1.97 ± 0.11	1.38 ± 0.15	2.35 ± 0.18
	Control	0.91 ± 0.11	0.93 ± 0.25	0.88 ± 0.14
AHO	Specimen	0.88 ± 0.12	0.53 ± 0.13	1.01 ± 0.16
	Control	0.56 ± 0.10	0.36 ± 0.13	0.77 ± 0.12

#### AHO

The AHO score differs significantly (*p* < 0.001) in the comparison of sick knees with healthy knees in the Mann-Whitney *U* test (see Fig. 4). The medial compartment also has a significantly different score than the lateral compartment. A comparison of m-covered and m-uncovered areas also shows a significant difference (*p* < 0.01). Looking at the diseased population in isolation, a difference between medial and lateral compartments can be observed in the Mann-Whitney *U* test. The m-covered areas also differ significantly from m-uncovered areas in terms of their AHO score. The AHO score correlates weaker negative with the cartilage thickness (− 0.14 Kendall-tau) and stronger negative with the IM.

#### Regression analysis

Kendall-tau shows slightly negative relationships between the two histological scores OARSI and AHO and the IM, as well as cartilage thickness. The *p* values consistently show significant results (see Table 5). The Pearson correlation coefficient shows slightly negative relationships between histological scores and IM, as well as cartilage thickness. Significant statements can be made for all tests except for the correlation between the AHO score and the cartilage thickness with *p* = 0.062.

**Table 5:** Overview of biomechanical and histological correlations

Correlation	Kendall-tau		Pearson-rho	
	Correlation coefficient	*p* value	Correlation coefficient	*p* value
OARSI/IM	− 0.37	6.2 × 10^−7^	− 0.33	6.1 × 10^−10^
AHO/IM	− 0.30	1.3 × 10^−3^	− 0.30	4.2 × 10^−7^
OARSI/thickness	− 0.26	4.0 × 10^−6^	− 0.11	0.013
AHO/thickness	− 0.14	0.016	− 0.08	0.062

## Discussion

### Automated mappings

#### Indentation

In this study a significant difference in the measured IM with *p* < 0.05 between the controls and the specimen was detected. Comparing the m-covered with the m-uncovered areas of the arthrotic cartilage, a significant difference could be observed. So far, there are very few studies dealing with the characterization of the mechanical properties of human joint cartilage [14–16]. Most studies are dealing with animal samples [17, 18]. There are no studies showing a complete mapping of the joint cartilage. Deneweth et al. [16] performed a mapping by punching out 21 cylindrical samples distributed over the tibia in a 4 × 3 grid and then examining them by unconfined compression. In comparison, we measured 100 ± 29 measuring points per tibia, depending on the samples. Compared with the study by Deneweth et al. [16] and with further studies which punched out three to four cylindrical samples on each tibia [15] and examined them by means of unconfined compression [19] or by means of Hayes’ indentation [37] in the m-covered and m-uncovered areas each with one measurement [21], significantly more measuring points per tibia can be examined by automated mapping. Of the measured values, the studies already published are in a similar range to our measurements, despite the different measurement methods. Thambyah et al. [21] discuss values between 2.13 ± 0.74 MPa (lateral, uncovered) and 5.13 ± 1.91 MPa (medial, covered) depending on the position (lateral, medial, m-covered, m-uncovered). Deneweth el al. [16] speak depending on the position of readings between 4.69 and 20.40 MPa for the lateral tibia and 7.01–30.83 MPa for the medial tibia. The novelty of this work, based on Sim et al. [22, 23], is the automatic examination of the entire articular cartilage of 38 human tibia without having to damage the tibia for the mechanical examinations.

#### Thickness measurement

The cartilage thickness of our measurements was within a range of 1.92 ± 0.89 mm for the m- covered areas and 2.03 ± 0.98 mm for the m-uncovered areas. Thambyah et al. [21] also investigated human tibiae with regard to cartilage thickness, m-covered, and m-uncovered. They reported cartilage thicknesses between 3.2–3.9 mm for the m-uncovered parts and between 1.7–2.1 mm for the m-covered areas. However, in Thambyah et al. [21], the samples came from significantly younger patients than in our study. In addition, all patients were male. In their MRI study, Faber et al. [42] describe cartilage thicknesses averaging 1.2–1.6 mm for female patients and 1.3–1.8 mm for male patients. All patients with 22 years were significantly younger than in our study with a mean of 72 years. Nevertheless, the values determined for cartilage thickness are in a similar range to the controls in our study. The study by Faber et al. [42] also did not differentiate between m-covered and m-uncovered, but only between medial and lateral. From the pure measured values, similar values were actually measured, similar to our study. For example, the following values were measured for the medial tibia: 1.90–4.06 mm female patients, 2.40–4.78 mm male patients.

### Histological examinations

The histological scores were 1.8 ± 1 of 6 for the OARSI score and 0.8 ± 0.8 of 3 for the AHO score. Chen et al. [43] showed similar values of 1.6 ± 0.3 for the ORASI score in a study with 20 histologically evaluated tibial plateaus in gonarthrosis. The higher the IM, the lower is the histological scores. Waldstein et al. [20] subjected cylindrical samples of 8 mm diameter to a dynamic investigation and showed that there is a correlation between the degree of arthrosis and the biomechanical measurements. A negative correlation could also be statistically demonstrated. Abedian et al. [26] also found a negative correlation (*r* = − 0.571, *p* = − 0.006) between the histological scores and the IM when examining a 60-year-old female’s knee. Aho et al. [41] showed a positive correlation between the AHO score and the OARSI score as well as a negative correlation between the AHO score and a low bone thickness in a study with 20 volunteers. Both the OARSI score (4.07 ± 1.31) and the AHO score (1.94 ± 0.99) were higher overall. Sampling in our study was performed only in areas where measurable cartilage was present for indentation. This meant that areas with bald bones, which would have achieved significantly higher scores, were not taken into account. This leads to an underestimation of the histological scores. This explains the relatively lower values in the histological scores in our study. On the other hand, biomechanical measurements cannot be performed at the defect sites where cartilage no longer exists. This would lead to an increase in the IM, as the bone would then be measured at these positions.

The weaknesses of the study are a different sample size than the controls. However, the specifications of the performance calculation were taken into account and even more than the minimum of 11 samples required for both specimen (25) and control (13) were used. Such laboratory tests are never fully comparable to in vivo conditions. The control is a population from the pathological institute. The age of the control is not known. However, it can be assumed that the age is advanced.

## Conclusions

In the presented study, the method for automatic mapping of IM and histological examination of the cartilage tissue in human tibiae showed a direct correlation between histological changes and altered biomechanics in gonarthrosis. In addition, the investigations showed that in the final stage of gonarthrosis, the damping behavior of the joint surface of the tibial plateau is significantly reduced compared to the healthy knee. In the future, a minimally invasive intraarticular indentation measurement may help us to decide between joint replacement and joint preserving therapy.

## Data Availability

Please contact the author for data requests.

## Abbreviations

IM: Instantaneous modulus
m-covered: Meniscus-covered
m-uncovered: Meniscus-uncovered
TKA: Total knee replacement
